# SARS-CoV-2 spike protein-mediated cardiomyocyte fusion may contribute to increased arrhythmic risk in COVID-19

**DOI:** 10.1371/journal.pone.0282151

**Published:** 2023-03-08

**Authors:** Daniel J. Clemens, Dan Ye, Wei Zhou, C. S. John Kim, David R. Pease, Chanakha K. Navaratnarajah, Alison Barkhymer, David J. Tester, Timothy J. Nelson, Roberto Cattaneo, Jay W. Schneider, Michael J. Ackerman

**Affiliations:** 1 Department of Molecular Pharmacology & Experimental Therapeutics, Windland Smith Rice Sudden Death Genomics Laboratory, Mayo Clinic, Rochester, MN, United States of America; 2 Division of Heart Rhythm Services, Department of Cardiovascular Medicine, Windland Smith Rice Genetic Heart Rhythm Clinic, Mayo Clinic, Rochester, MN, United States of America; 3 Discovery Engine/Program for Hypoplastic Left Heart Syndrome, Mayo Clinic, Rochester, MN, United States of America; 4 Department of Molecular Medicine, Mayo Clinic, Rochester, MN, United States of America; 5 Wanek Family Program for HLHS-Stem Cell Pipeline, Mayo Clinic, Rochester, MN, United States of America; 6 Division of Pediatric Cardiology, Department of Pediatric and Adolescent Medicine, Mayo Clinic, Rochester, MN, United States of America; The Open University, UNITED KINGDOM

## Abstract

**Background:**

SARS-CoV-2-mediated COVID-19 may cause sudden cardiac death (SCD). Factors contributing to this increased risk of potentially fatal arrhythmias include thrombosis, exaggerated immune response, and treatment with QT-prolonging drugs. However, the intrinsic arrhythmic potential of direct SARS-CoV-2 infection of the heart remains unknown.

**Objective:**

To assess the cellular and electrophysiological effects of direct SARS-CoV-2 infection of the heart using human induced pluripotent stem cell-derived cardiomyocytes (hiPSC-CMs).

**Methods:**

hiPSC-CMs were transfected with recombinant SARS-CoV-2 spike protein (CoV-2 S) or CoV-2 S fused to a modified Emerald fluorescence protein (CoV-2 S-mEm). Cell morphology was visualized using immunofluorescence microscopy. Action potential duration (APD) and cellular arrhythmias were measured by whole cell patch-clamp. Calcium handling was assessed using the Fluo-4 Ca^2+^ indicator.

**Results:**

Transfection of hiPSC-CMs with CoV-2 S-mEm produced multinucleated giant cells (syncytia) displaying increased cellular capacitance (75±7 pF, n = 10 vs. 26±3 pF, n = 10; *P*<0.0001) consistent with increased cell size. The APD90 was prolonged significantly from 419±26 ms (n = 10) in untransfected hiPSC-CMs to 590±67 ms (n = 10; *P*<0.05) in CoV-2 S-mEm-transfected hiPSC-CMs. CoV-2 S-induced syncytia displayed delayed afterdepolarizations, erratic beating frequency, and calcium handling abnormalities including calcium sparks, large “tsunami”-like waves, and increased calcium transient amplitude. After furin protease inhibitor treatment or mutating the CoV-2 S furin cleavage site, cell-cell fusion was no longer evident and Ca^2+^ handling returned to normal.

**Conclusion:**

The SARS-CoV-2 spike protein can directly perturb both the cardiomyocyte’s repolarization reserve and intracellular calcium handling that may confer the intrinsic, mechanistic substrate for the increased risk of SCD observed during this COVID-19 pandemic.

## Introduction

Since its discovery in the Wuhan province of China at the end of 2019, severe acute respiratory syndrome coronavirus 2 (SARS-CoV-2), the virus responsible for the coronavirus disease 2019 (COVID-19) pandemic, has claimed the lives of over 800,000 individuals in the United States alone, and over 5.5 million individuals worldwide [1, 2]. Most complications in patients with COVID-19 are due to pulmonary injury which leads to respiratory failure.

However, it is now well established that SARS-CoV-2 infection can lead to a myriad of cardiovascular complications including severe arrhythmias and sudden cardiac death (SCD) [3, 4]. In fact, multiple studies of the early stages of the COVID-19 pandemic in New York City, Italy, and Paris illuminated the significantly higher rates of out of hospital cardiac arrest compared to previous years [5–7]. Multiple intrinsic, environmental, and COVID-19-specific risk factors may be leading to increased risk of potentially fatal arrhythmias including thrombosis, an exaggerated immune response, the presence of high-risk co-morbidities, and initial treatment with QT prolonging drugs such as hydroxychloroquine and azithromycin early in the pandemic [8].

In addition to these risk factors is the potential for direct SARS-CoV-2 infection of the heart. Albeit rarely, this has been detected to a variable extent in numerous biopsy and autopsy studies of human myocardium from patients with COVID-19 [9–17]. Direct SARS-CoV-2 infection of ACE2-expressing human cardiomyocytes has also been observed in multiple *in vitro* cardiac cell models [18–22]. Most recently, Navaratnarajah et al. showed that efficient and productive SARS-CoV-2 infection of human induced pluripotent stem cell-derived cardiomyocytes (hiPSC-CMs) leads to the formation of multinucleated giant cells called syncytia through cell-cell fusion which is mediated by the SARS-CoV-2 spike protein [23]. However, despite this evidence for direct cardiac damage by SARS-CoV-2, little is known about the arrhythmic potential this may incur, and greater clarification, particularly at the level of the cardiomyocyte, is needed in order to better understand this critically important aspect of COVID-19 pathobiology.

Therefore, in this study, we determined the electrophysiological effects, cytosolic calcium handling, and arrhythmic potential, of direct SARS-CoV-2 infection of the heart in the ACE2-accentuated, bio-engineered hiPSC-CM model system.

## Methods and materials

Control human fibroblast-derived hiPSCs were obtained under Mayo Clinic IRB approved protocol. Detailed spinner culture cardiac differentiation protocol was described previously [23]. Differentiated hiPSC-CMs were plated on fibronectin-coated glass coverslips and maintained in Gibco™ Cardiomyocyte Maintenance Medium. hiPSC-CMs were transfected with either recombinant SARS-CoV-2 spike protein (CoV-2 S) or CoV-2 S fused to a modified Emerald fluorescence protein (CoV-2 S-mEm). Cell morphology was visualized using light and confocal immunofluorescence microscopy. Action potential duration (APD) and cellular arrhythmias were measured by whole cell patch-clamp. Calcium handling was assessed by live cell imaging using the Fluo-4 Ca^2+^ indicator.

### Spinner culture cardiac differentiation of human induced pluripotent stem cells (hipscs)

Control human fibroblast-derived hiPSCs were obtained under Mayo Clinic IRB approved protocol and maintained in mTESR1 basal media with mTESR supplement on Geltrex (in DMEM/F12 media) coated plates. Undifferentiated hiPSCs were transitioned and expanded in suspension/spinner culture in DMEM/F-12 plus Glutamax, StemPro supplement, BSA and bFGF with Rock Inhibitor Y27632 combined with mTESR1 media, and then differentiated using a previously established protocol (CHIR/IWP-4) into CMs in RPMI 1640 plus B27 minus insulin supplement as beating aggregates. Detailed spinner culture cardiac differentiation protocol is available from J.W.S. upon request. Differentiated hiPSC-CMs were plated on fibronectin-coated glass coverslips and maintained in Gibco™ Cardiomyocyte Maintenance Medium.

### Plasmids and mutagenesis

The codon-optimized SARS-CoV-2 spike protein (CoV-2 S) gene (YP_009724390) was synthesized by Genewiz in a pUC57-Amp plasmid. The CoV-2 S coding sequence was cloned into a pCG mammalian expression plasmid [24] using unique restriction sites *Bam*HI and *Spe*I. CoV-2 S was tagged with a modified Emerald reporter (CoV-2 S-mEm) by cloning the mEm sequence (Addgene, Plasmid #53976) to the C-terminal end of the SARS CoV-2 S protein in the pCG expression vector. A flexible 6 amino acid-linker (TSGTGG) was used to separate the two proteins. All expression constructs were verified by sequencing the entire coding region. QuikChange site-directed mutagenesis (Agilent Technologies, Santa Clara, CA) was used to insert the R682S furin cleavage variant into the CoV-2 S expression plasmid. Two independent clones were verified by sequencing the CoV-2 S gene at the location of the variant.

### Transfection of hiPSC-CMs

Day-20 hiPSC-CMs were plated in a subconfluent monolayer on fibronectin-coated glass coverslips in 6-well plates and transfected with 1–2 μg plasmid using Lipofectamine 3000. Following transfection of hiPSC-CMs with CoV-2 S-mEm or CoV-2 S, syncytia formation became obvious within 6 hours of transfection. For furin inhibitor experiments, 20 μM Furin Inhibitor I (Decanoyl-RVKR-CMK, Calbiochem, #344930) [23] was dissolved in DMSO and added to hiPSC-CMs maintenance media 2-hours after transfection. Furin inhibitor was removed prior to the calcium imaging experiments.

### Immunofluorescence microscopy

hiPSC-CMs were fixed with 4% paraformaldehyde for 10 minutes at room temperature (RT). After fixing, cells were washed 3 times with PBS, permeabilized/blocked with PBS containing 0.1% Triton X-100 (PBST)/5% goat serum for 1 hour at RT, then incubated overnight in primary antibody solution made of PBST/5% goat serum containing primary antibody against α-Actinin (1:500 dilution, mouse, Sigma, A7811), calreticulin (1:200 dilution, rabbit, Abcam, ab2907) or SERCA2a (rabbit, Cell Signaling, 4388S). The following day, cells were washed 3 times with PBST at RT before being incubated in PBST/5% goat serum with a 1:250 dilution of Alexa Fluor 488 goat-anti-mouse (Thermo Fisher Scientific, A11001) and Alexa Fluor 594 goat-anti-rabbit (Thermo Fisher Scientific, A11037) secondary antibodies at RT for 1 hour. After secondary antibody incubation, cells were washed 3 times with PBST. After air drying, cells were mounted in anti-fade medium with DAPI (Vector Laboratories, H-1000). Images were acquired on a Zeiss LSM 780 confocal microscope (Oberkochen, Germany) in Mayo Clinic’s Microscopy and Cell Analysis Core.

### Electrophysiology

Action potentials (APs) from untransfected or SARS-CoV-2 S-mEmerald transfected hiPSC-CMs were recorded at RT (22–24°C) using current clamp mode at 1 Hz (8 cells) and 0.67 Hz (2 cells with very long APD) through 5 ms depolarizing current injections of 300–500 pA and gap free configuration with an Axopatch 200B amplifier, Digidata 1440A and pClamp version 10.4 software. Untransfected single hiPSC-CMs and SARS-CoV-2S-mEmerald transfected hiPSC-CMs with brighter green fluoresce (syncytia) were selected for AP measurements. The extracellular (bath) solution contained (mmol/L): 150 NaCl, 5.4 KCl, 1.8 CaCl_2_, 1 MgCl_2_, 1 Na-Pyruvate and 15 HEPES, pH adjusted to 7.4 with NaOH. The pipette solution contained (mmol/L): 150 KCl, 5 NaCl, 2 CaCl_2_, 5 EGTA, 5 MgATP and 10 HEPES, pH adjusted to 7.2 with KOH [25]. Data were analyzed using Clampfit and Excel (Microsoft, Redmond, WA), and graphed with GraphPad Prism 8.3 (GraphPad Software, San Diego, CA). All data points are shown as the mean value and bars represent the standard error of the mean. An unpaired two-tailed Student’s t-test was performed to determine statistical significance between two groups. A *P*<0.05 was considered to be significant.

### Calcium imaging

Untransfected and SARS-CoV-2 S transfected hiPSC-CMs cultured on fibronectin-coated 35mm glass-bottom dishes (MatTek Corporation, Ashland, MA) at 37°C, 5% CO_2_ were loaded with 5μM of Fluo-4 AM (Thermo Fisher Scientific, Waltham, MA) with 0.02% F-127 (Thermo Fisher Scientific, Waltham, MA) in Tyrode’s Solution (Alfa Aesar, Tewksbury, MA) for 30 minutes. Following wash-out, Tyrode’s solution was added, and cells were imaged. During imaging, cells were kept in a heated 37°C stage-top environment chamber supplied with 5% CO_2_. Imaging of Ca^2+^ transients was taken under a 40X objective using a Nikon Eclipse Ti (Melville, NY) light microscope. Human iPSC-CMs were paced at 1 Hz using an IonOptix MyoPacer Field Stimulator (Westwood, MA). Time-lapse videos were taken at a speed of 20ms per frame for 20s. Each video recording was analyzed for the percent area exhibiting pacing, calcium sparks, and “calcium tsunamis”. The raw data was exported to Excel software (Microsoft, Redmond, WA) and analyzed with a custom Excel-based program in order to normalize for photo bleaching and movement. All values are reported as mean ± SEM. Statistical analysis was performed using GraphPad Prism 8 software (San Diego, CA). An unpaired two-tailed Student’s t-test was used to determine statistical significance between two groups, and a one-way ANOVA followed by Tukey’s multiple comparisons test was used to determine statistical significance between 3 groups. A *P* <0.05 was considered to be significant.

## Results

### SARS-CoV-2 spike protein-mediated cardiomyocyte fusion

**Fig 1:**
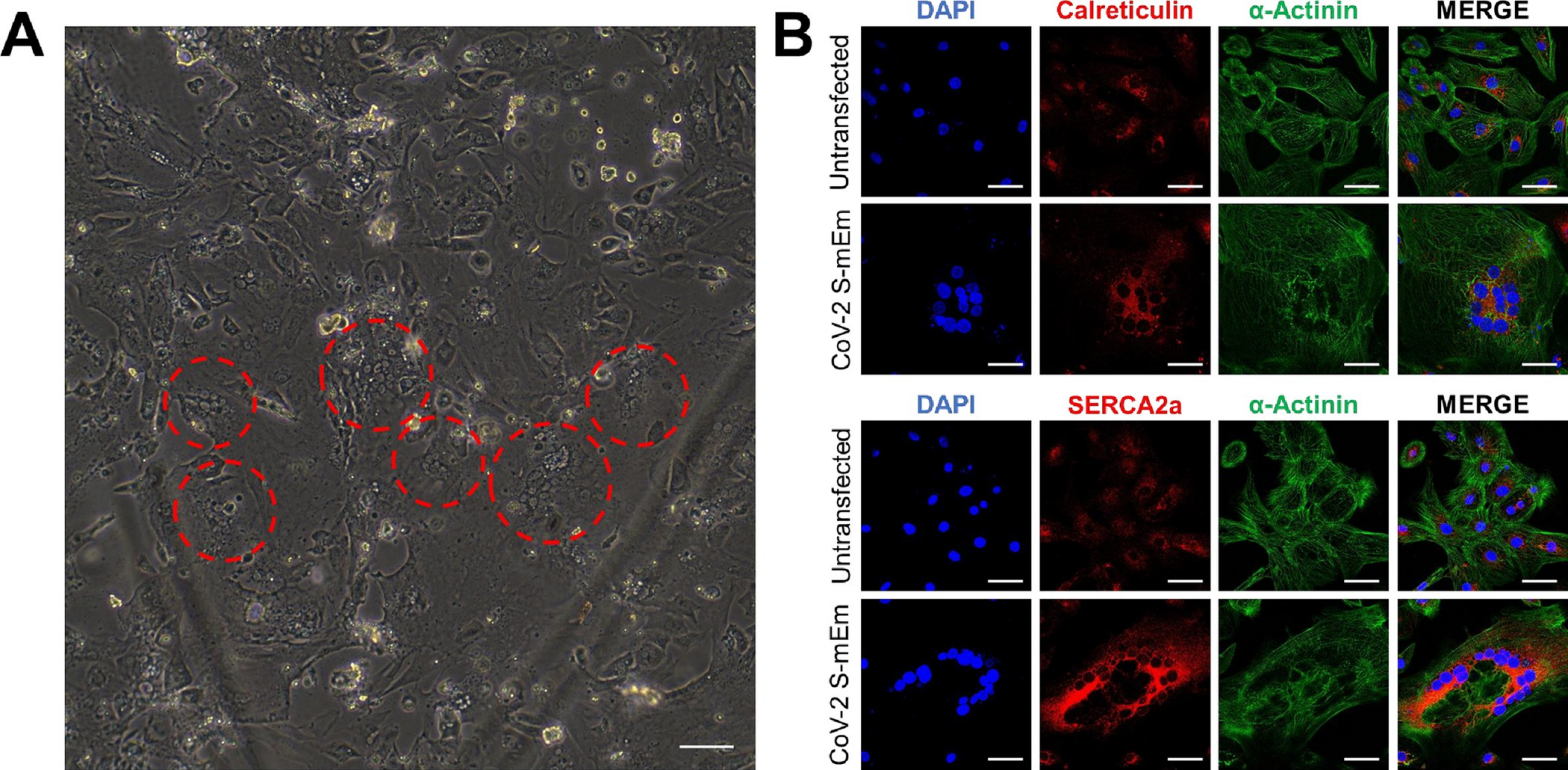
SARS-CoV-2 spike protein induces formation of syncytia in hiPSC-CMs. (**A**) Light microscopy showing hiPSC-CMs following transfection with CoV-2 S-mEm. Red circles indicate clusters of nuclei which represent hiPSC-CM syncytia. Scale bar 400 μm. (**B**) Confocal immunofluorescence microscopy of both untransfected and CoV-2 S-mEm-transfected hiPSC-CMs displaying the cardiac marker α-Actinin and sarcoplasmic reticulum proteins calreticulin and SERCA2a. Scale bars 50 μm.

To study the potential electrophysiological effects of SARS-CoV-2 infection in human cardiomyocytes, we transfected our bio-engineered hiPSC-CM model system with full-length recombinant SARS-CoV-2 spike protein fused at its C-terminal end to a modified Emerald green fluorescent protein (CoV-2 S-mEm). As documented previously with infection of hiPSC-CMs with the entire live virus [23], within just 6 hours post transfection, we began to observe the formation of syncytia resulting from CoV-2 S-mEm-mediated cell-cell fusion. **Fig 1A** documents several giant cells with central clusters of nuclei, typical virus glycoprotein-mediated syncytia (indicated by dotted red lines). These syncytia were identified by large clusters of many nuclei and were visible by light microscopy [23]. This phenomenon could be more clearly seen by immunofluorescence confocal microscopy images of both untransfected and CoV-2 S-mEm transfected hiPSC-CMs stained with antibodies against the cardiac marker α-Actinin and multiple sarcoplasmic reticulum (SR)-related proteins (calreticulin and SERCA2a; **Fig 1B**).

In order to confirm syncytia formation was caused by spike protein itself, we transfected iPSC-CMs with Mock (empty vector) or CoV-2 S and compared the percentage of syncytia formation in each group using calcium indicator Fluo-4. We found that 25% (67/268 cells) of CoV-2 S transfected iPSC-CMs showed syncytia formation which was significantly different from that in untransfected iPSC-CMs (2 /170 cells, 1.2%, p<0.0001), Mock iPSC-CMs (1/122 cells, 0.8%, p<0.0001), but there was no difference between untransfected (1.2%) and Mock iPSC-CMs (0.8%, p = 0.74, **S1 Fig, S1–S5 Videos**). Our data suggests that syncytia formation of iPSC-CMs is associated with CoV-2 S only. Mock transfection with an empty vector did not cause cell fusion.

### Transfection with SARS-CoV-2 spike protein induces action potential prolongation and cellular arrhythmias

**Fig 2:**
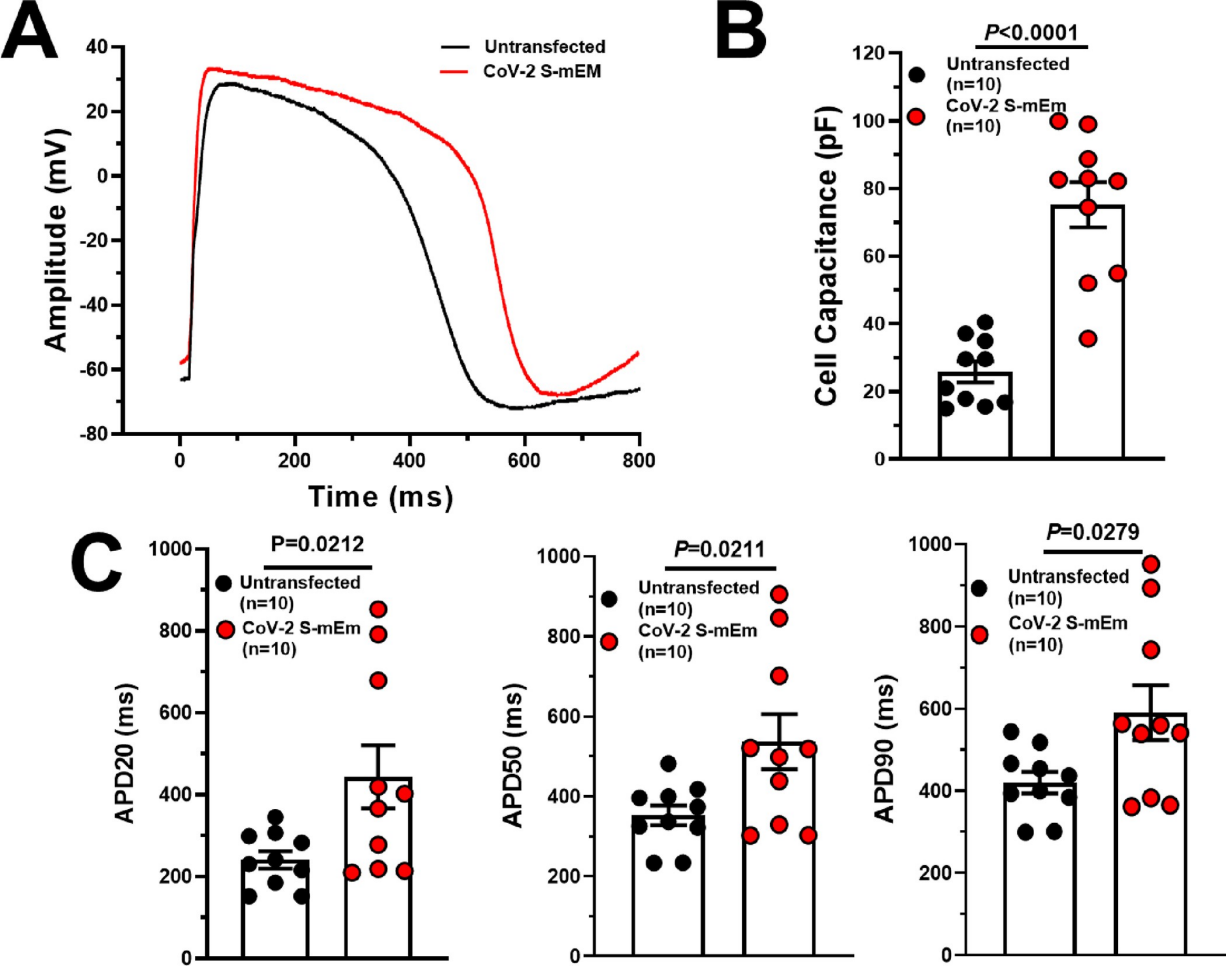
SARS-CoV-2 spike protein-induced hiPSC-CM syncytia exhibit reduced repolarization reserve with marked prolongation in action potential duration (APD). (**A**) Representative action potential traces derived from untransfected and CoV-2 S-mEm-transfected hiPSC-CMs. (**B**) Cell capacitance in untransfected (n = 10) and CoV-2 S-mEm-transfected (n = 10) hiPSC-CMs. (**C**) Action potential duration at 20% (APD20; left), 50% (APD50; middle) and 90% (APD90; right) in untransfected hiPSC-CMs (n = 10) paced at 1 Hz and CoV-2 S-mEm-transfected hiPSC-CMs paced at 1 Hz (8 cells) and 0.67 Hz normalized to 1 Hz (2 cells). Graphs are presented as mean±SEM. Unpaired two-tailed Student’s t-tests were conducted to determine significance.

We next utilized standard whole cell patch-clamp technique to assess the electrophysiological phenotype of the CoV-2 S-mEm-induced hiPSC-CM syncytia (refer to **Fig 1A** for the visual definition of syncytia). Cellular capacitance, which is an indirect measure of cell size, was significantly increased in CoV-2 S-mEm transfected hiPSC-CMs compared to untransfected control (75±7 pF, n = 10 vs. 26±3 pF, n = 10, *P*<0.0001; **Fig 2B**) indicating that the hiPSC-CMs have indeed fused together to form one large syncytium following transfection with CoV-2 S-mEm.

The action potential duration at 20% (APD20), 50% (APD50) and 90% (APD90) repolarization was prolonged significantly in CoV-2 S-mEm-induced syncytia paced at 1Hz (8 cells) or 0.67 Hz normalized to 1Hz (2 cells)(APD20: 443±77 ms, n = 10 vs. 241±21 ms, n = 10, P = 0.0212; APD50: 536±69 ms, n = 10 vs. 352±25 ms, n = 10, *P* = 0.0211; APD90: 590±67 ms, n = 10 vs. 419±26, n = 10, *P* = 0.0279; **Fig 2A and 2C**). It was also notable that the APD in hiPSC-CMs transfected with CoV-2 S-mEM was extremely variable with measurements from 361 ms to 951 ms (range = 590 ms), a range more than twice that observed in untransfected hiPSC-CMs (299–544 ms; range = 245 ms; **Fig 2C**). Resting membrane potential (RMP), maximal diastolic potential (MDP) and maximal upstroke velocity (dV/dtmax) did not reach statistically significant difference between transfected and untransfected cells. Only action potential amplitude (Amp) revealed statistically significant between two group (**S1 Table**).

**Fig 3:**
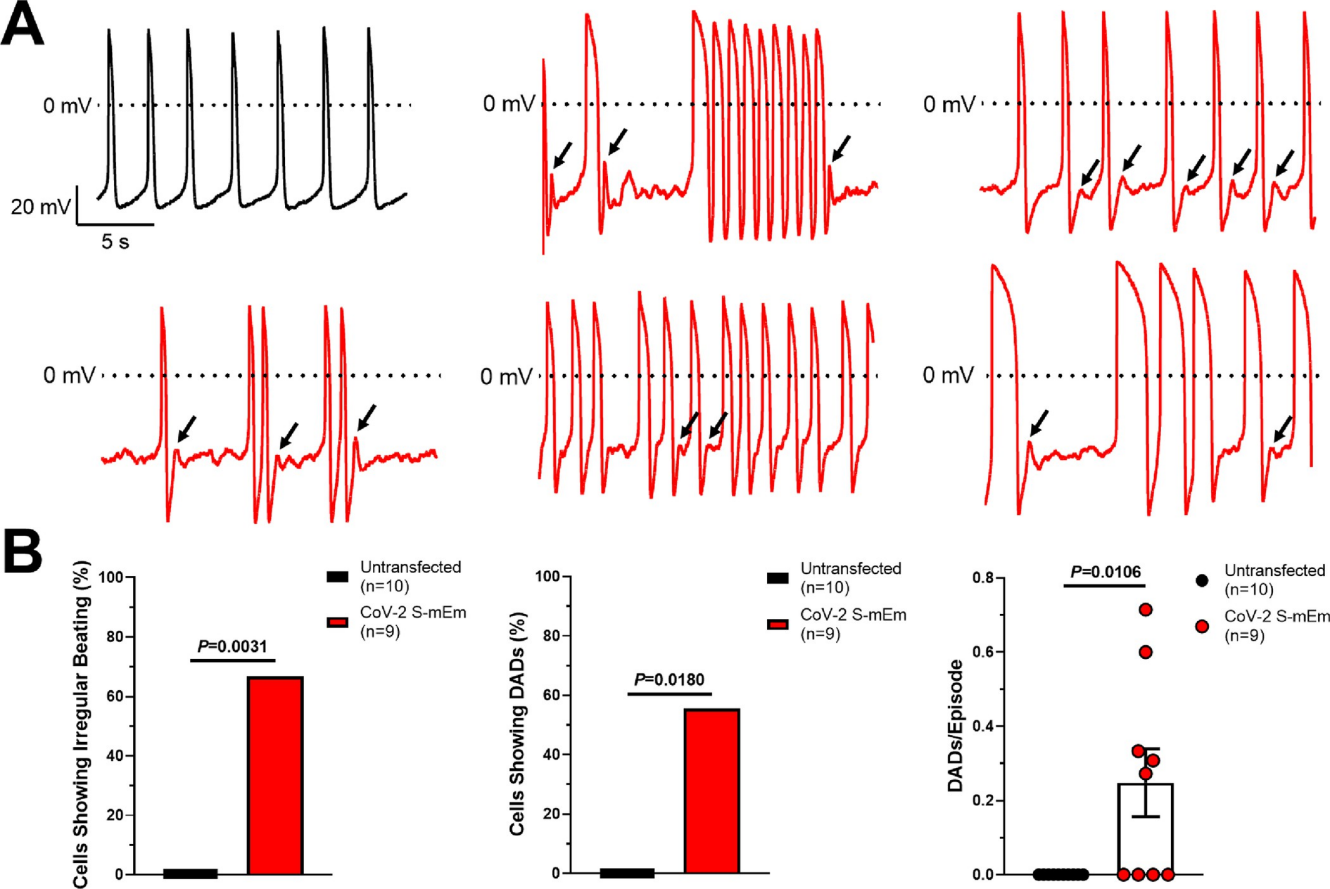
SARS-CoV-2 spike protein-induced hiPSC-CM syncytia display cellular arrhythmias. (**A**) Representative action potential traces derived from untransfected hiPSC-CMs showing regular beating (black) and CoV-2 S-mEm-transfected hiPSC-CMs displaying erratic beating and delayed afterdepolarizations (DADs; red). Black arrows indicate individual DADs. (**B**) Quantification of the number of untransfected (n = 10) or CoV-2 S-mEm-transfected (n = 9) hiPSC-CMs showing irregular beating (left) or delayed afterdepolarizations (DADs; middle) as well as the rate at which DADs occur (DADs/episode; right). Graphs depicting categorical data are presented as percentage, and Fischer’s exact tests were used to determine significance. Graphs depicting continuous data are presented as mean±SEM, and unpaired two-tailed Student’s t-tests were used to determine significance.

Additionally, CoV-2 S-mEm-induced syncytia displayed extremely irregular arrhythmic activity during spontaneous beating (**Fig 3A**). Overall, we observed irregular beating in 6/9 (66.6%) CoV-2 S-mEm transfected hiPSC-CMs compared to 0/10 (0%, *P* = 0.0031) untransfected hiPSC-CMs (**Fig 3B**). These beating irregularities varied greatly from one syncytium to the next as seen clearly in **Fig 3A**. Not only was beating frequency irregular, but in some recordings, the APD varied significantly from one action potential to the next which is extremely abnormal. CoV-2 S-mEm-induced syncytia also displayed frequent delayed afterdepolarizations (DADs). DADs were observed in 5/9 (55.5%) CoV-2 S-mEm-expressing cells and 0/10 (0%, *P* = 0.0108) untransfected hiPSC-CMs (**Fig 3B**). The rate at which these DADs occurred (DADs/Episode) was also significantly higher in CoV-2 S-mEm transfected hiPSC-CMs compared to untransfected control (0.25±0.09, n = 9, vs. 0.00±0.00, n = 10, *P* = 0.0106; **Fig 3B**).

### Transfection with SARS-CoV-2 spike protein can cause calcium mishandling

**Fig 4:**
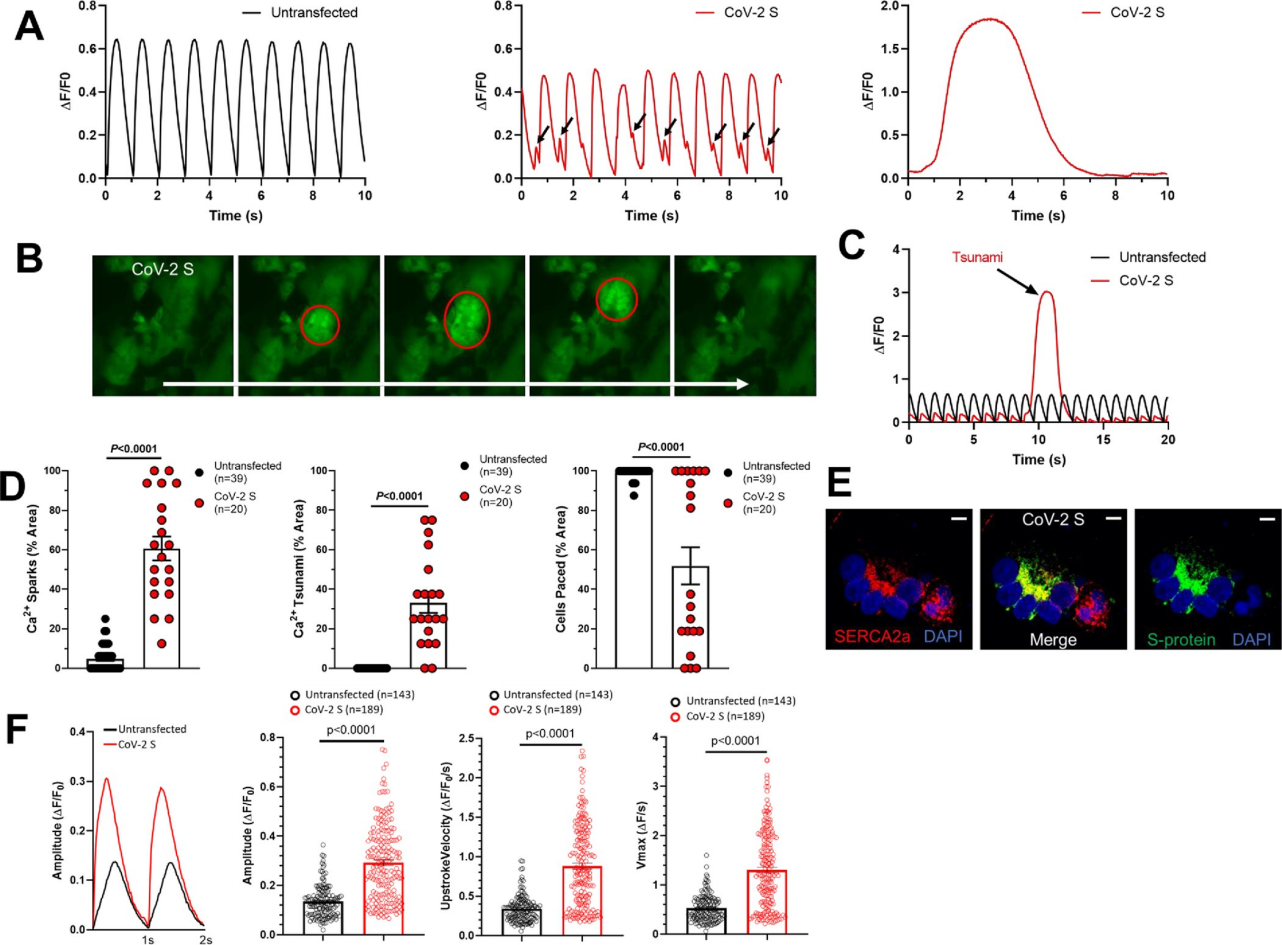
SARS-CoV-2 spike protein-induced hiPSC-CM syncytia display abnormal calcium handling. (**A**) Representative Fluo-4 calcium imaging traces derived from untransfected hiPSC-CMs (black) showing normal calcium transients (left) and from CoV-2 S-transfected hiPSC-CMs (red) showing calcium sparks (middle) and tsunamis (right). (**B**) Still frame images of Fluo-4 calcium imaging in CoV-2 S-transfected hiPSC-CMs. Red circles indicates the calcium tsunami. (**C**) Representative Fluo-4 calcium imaging traces comparing the amplitude and duration of a calcium tsunami in CoV-2 S-transfected hiPSC-CMs to normal calcium transients in untransfected hiPSC-CMs. (**D**) Quantification of calcium sparks (left), calcium tsunamis (middle), and pacing (right) in untransfected (n = 39) and CoV-2 S-transfected (n = 20) hiPSC-CMs. (**E**) Confocal immunofluorescence microscopy showing colocalization of SERCA2a and CoV-2 S. Scale bars 10 μm. (**F**) Representative Fluo-4 calcium imaging traces and data summary graphs derived from untransfected hiPSC-CMs (black, n = 143) showing normal calcium transients and from CoV-2 S-transfected hiPSC-CMs (red, n = 189) showing increased amplitude, upstroke velocity and Vmax of calcium transient. Graphs are presented as mean±SEM. Unpaired two-tailed Student’s t-tests were conducted to determine significance.

Because DADs are often caused by abnormal or spontaneous SR calcium release, we next performed Fluo-4 calcium imaging measurements in both untransfected and CoV-2 S (no modified Emerald fluorescent protein)-transfected hiPSC-CMs. As expected, untransfected CMs displayed consistent and rhythmic calcium transients when paced at 1 Hz. CoV-2 S-induced syncytia, however, displayed multiple calcium handling abnormalities including calcium sparks, which are known to be an underlying cause of DADs and a novel phenomenon which we have termed “calcium tsunamis” (**Fig 4A**).

These calcium tsunamis are large calcium waves which begin in one area and slowly move throughout the syncytia. Additionally, these observed calcium tsunamis have a significantly greater amplitude and duration than normal calcium transients. This can be seen clearly from the still frame images and representative traces in **Fig 4A–4C**, but is best appreciated by video microscopy (**S1–S5 Videos**).

Since calcium tsunamis occur in syncytia, we quantified calcium handling abnormalities by measuring the % area of each video recording. Both calcium sparks and calcium tsunamis were significantly more common in CoV-2 S-transfected hiPSC-CMs compared to untransfected control (Sparks: 64.6±6.0%, n = 20, vs. 4.8±1.0%, n = 39, *P*<0.0001; Tsunamis: 33.1±5.1%, n = 20, vs. 0.0±0.0%, n = 39, *P*<0.0001; **Fig 4D**). Additionally, we observed a loss of beating in some CoV-2 S-induced syncytia, despite 1 Hz electrical pacing, which would indicate a complete loss of function leading to beating arrest. This was significantly more common in CoV-2 S-induced syncytia compared to untransfected hiPSC-CMs (Cells Paced: 51.9±9.4%, n = 20, vs. 99.0±0.4%, *P*<0.0001; **Fig 4D**). Finally, the transfected CoV-2 S protein co-localized with SERCA2a at the sarcoplasmic reticulum which may contribute to the severe calcium mishandling observed in CoV-2 S-transfected hiPSC-CMs (**Fig 4E**).

Single cell calcium handling analysis of untransfected and CoV-2 S-transfected hiPSC-CMs showed increased amplitude, upstroke velocity and Vmax of calcium transient (**Fig 4F**). The calcium transient amplitude normalized by (ΔF/F0) was significantly increased in CoV-2 S-transfected hiPSC-CMs compared to untransfected hiPSC-CMs (untransfected: 0.14±0.01, n = 143; CoV-2 S-transfected: 0.29±0.01, n = 189, p<0.0001; **Fig 4F**). The CoV-2 S- transfected hiPSC-CMs displayed a significantly faster upstroke velocity compared to untransfected hiPSC-CMs (untransfected: 0.34±0.01, n = 143; CoV-2 S-transfected: 0.88±0.04, n = 189, p<0.0001; **Fig 4F**). The CoV-2 S- transfected hiPSC-CMs displayed a significant increase in Vmax of calcium transient compared to untransfected hiPSC-CMs (untransfected: 0.53±0.02, n = 143; CoV-2 S-transfected: 1.31±0.06, n = 189, p<0.0001; **Fig 4F**).

### Biochemical and genetic interference prevents SARS-CoV-2 spike protein-mediated fusion and normalizes calcium handling

In the study by Navaratnarajah et al., the authors showed that cell-cell fusion could be prevented by treatment with either i) a cell-permeable, peptide-based molecule known as Decanoyl-RVKR-CMK (furin inhibitor, FI) which irreversibly blocks the catalytic site of a protease in the Golgi called furin or ii) genetic interference by inserting a single amino acid change, R682S, which is expected to inactivate the furin cleavage site of CoV-2 S [23]. Knowing this, we sought to test whether these same treatments could prevent the abnormal calcium handling observed in CoV-2 S-induced syncytia in our reduced, bio-engineered cardiomyocyte model system.

**Fig 5:**
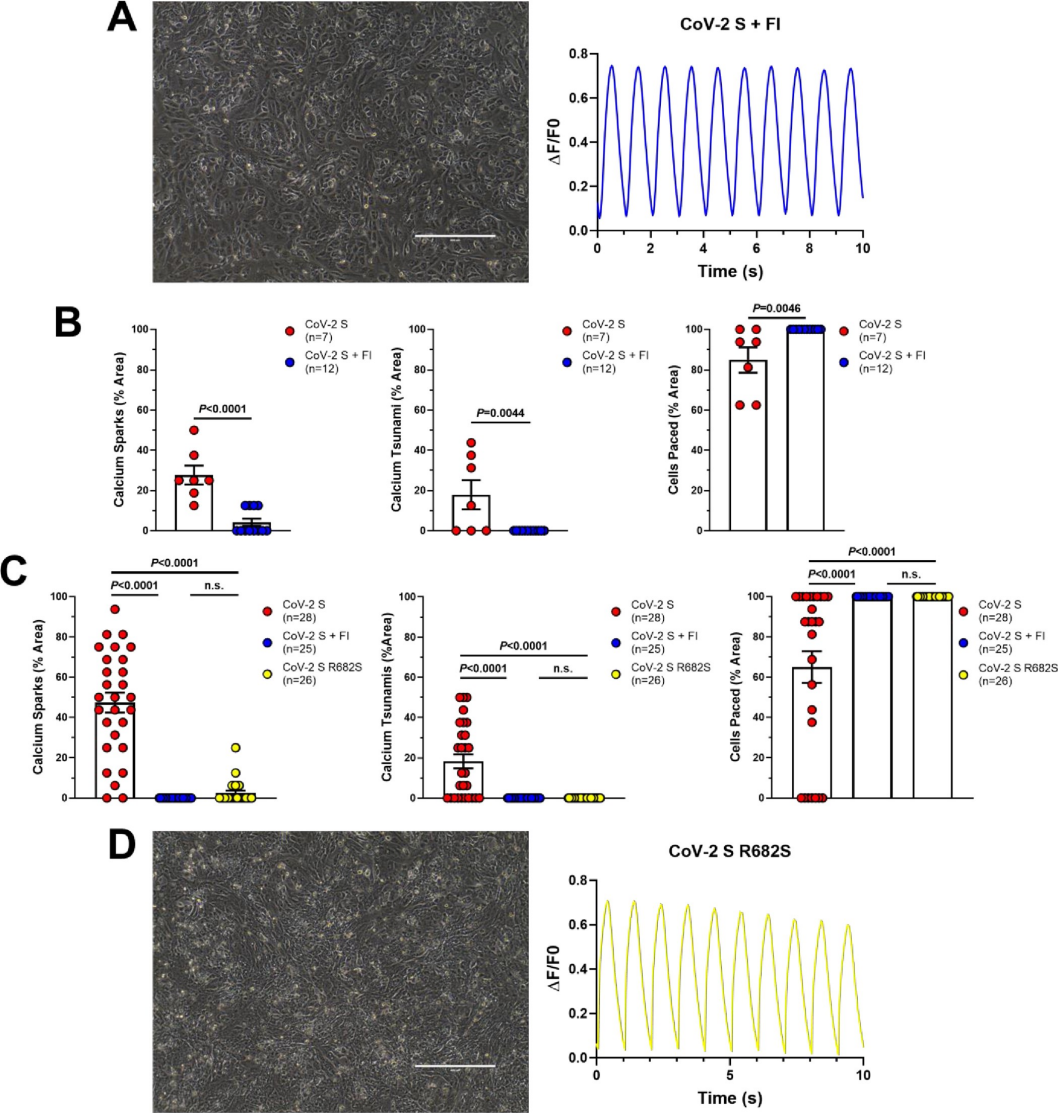
Biochemical and genetic interference prevents formation of hiPSC-CM syncytia and restores calcium handling. (**A**) Light microscopy (left) and representative Fluo-4 calcium imaging trace (right) from CoV-2 S-transfected hiPSC-CMs treated with furin inhibitor (FI). Scale bar 400 μm. (**B**) Quantification of calcium sparks (left), calcium tsunamis (middle), and pacing (right) in CoV-2 S-transfected (n = 7) and CoV-2 S + FI (n = 12) hiPSC-CMs. (**C**) Quantification of calcium sparks (left), calcium tsunamis (middle), and pacing (right) in a second line of hiPSC-CMs transfected with CoV-2 S (n = 28), CoV-2 S + FI (n = 25), and CoV-2 S R682S (n = 26). (**D**) Light microscopy (left) and representative Fluo-4 calcium imaging trace (right) from CoV-2 S R682S-transfected hiPSC-CMs. Scale bar 400 μm. Graphs are presented as mean±SEM. Unpaired two-tailed Student’s t-tests were conducted to determine significance between two groups and one-way ANOVAs with Tukey’s multiple comparisons tests were conducted to determine significance between three groups.

In line with the previous findings, blockage of furin with FI prevented syncytia formation in hiPSC-CMs transfected with only CoV-2 S (**Fig 5A**). Prevention of cell-cell fusion using this treatment also led to normalization of calcium handling, including a significant reduction in calcium sparks (27.7±4.7%, n = 7, vs. 4.2±1.8%, n = 12, *P*<0.0001), complete absence of calcium tsunamis (17.9±7.3%, n = 7, vs. 0.0±0.0%, n = 12, *P* = 0.0044), and prevention of beating arrest in CoV-2 S-transfected hiPSC-CMs (84.8±6.2%, n = 7, vs. 100.0±0.0, n = 12, *P* = 0.0046; **Fig 5A and 5B**).

To confirm that treatment with FI can prevent calcium mishandling in hiPSC-CMs transfected with CoV-2 S, these studies were replicated in a second hiPSC line. Prior to FI treatment, we again observed calcium sparks, calcium tsunamis, and beating arrest in CoV-2 S-transfected hiPSC-CMs. As before, all of these abnormalities were eliminated completely by treatment with FI (**Fig 5C**).

Finally, hiPSC-CMs transfected with the engineered R682S mutation-containing spike protein did not form syncytia and displayed normal calcium handling properties similar to CoV-2 S-transfected hiPSC-CMs treated with FI (**Fig 5C and 5D**).

## Discussion

Over the course of the COVID-19 pandemic, it has become clear that SARS-CoV-2 infection can precipitate, albeit rarely thankfully, significant cardiac complications and potentially lethal arrhythmias [3–7]. There are many potential pro-arrhythmic substrates for patients with COVID-19, but a primary, intrinsic perturbation in cardiac excitation-contraction coupling and repolarization reserve conferring increased host vulnerability has been difficult to characterize mechanistically. Therefore, the bio-engineered hiPSC-CMs provides a reduced model system to directly study the potential effects of SARS-CoV-2 infection in human heart cells.

Regardless of the limitations of iPSC-derived cardiac myocytes as representative of the adult cell phenotype [26], to date, multiple studies of SARS-CoV-2 infection in hiPSC-CMs have revealed novel insights into COVID-19 pathobiology. These investigations have established that hiPSC-CMs can be infected directly with SARS-CoV-2 and display efficient viral reproduction [18–23]. Interestingly, while ACE2 (the receptor that SARS-CoV-2 spike protein hijacks to access cellular entry) is highly expressed in hiPSC-CMs, the protease TMPRSS2 is very lowly expressed suggesting an alternative route of SARS-CoV-2 infection in cardiomyocytes, potentially through cathepsin proteases [19, 20, 22, 23]. As such, observations from this reduced, ACE2-accentuated model system may reflect potential pro-arrhythmic susceptibility, not in the normal, healthy host, but rather in the patient who has other co-morbidities that trigger up-regulation in ACE2 levels in the heart such as hypertension and hypertrophic cardiomyopathy [27, 28]. Additionally, exposure to SARS-CoV-2 led to significant disruption of normal hiPSC-CM structure either through transcriptional changes or, as reported recently by Navaratnarajah et al., through the formation of syncytia following cell-cell fusion [20, 23].

To date, only two other studies have assessed how SARS-CoV-2 impacts hiPSC-CM function [21, 22]. Given the lack of functional data, we sought to better characterize the electrophysiological and arrhythmic effects of SARS-CoV-2 infection in hiPSC-CMs. Here, we observed that CoV-2 S-mEm (spike protein)-transfected hiPSC-CMs displayed increased cell capacitance indicating these cells had fused together to form one large syncytia, thus confirming the findings by Navaratnarajah et al. that expression of SARS-CoV-2 spike protein in hiPSC-CMs leads to the formation of syncytia by cell-cell fusion [23]. Additionally, we discovered that CoV-2 S-mEm-induced hiPSC-CMs syncytia displayed an extremely arrhythmic phenotype including erratic beating frequency and frequent DADs.

One potential underlying cause of these cellular arrhythmias was abnormal calcium handling. Following transfection with CoV-2 S, hiPSC-CMs displayed frequent calcium sparks. These small, spontaneous calcium release events are well known to be an underlying cause of DADs and ventricular arrhythmias in patients with underlying genetic heart disease such as catecholaminergic polymorphic ventricular tachycardia for example [29, 30], and are therefore the likely cause of the DADs observed by patch clamp in CoV-2 S-mEm-induced syncytia. Interestingly, Dimai et al. recently observed similar spontaneous calcium release events in otherwise normal hiPSC-CMs that were incubated in serum extracted from SARS-CoV-2 positive patients who were hospitalized for severe acute respiratory distress [31].

In addition to calcium sparks, we observed a novel calcium handling phenomenon which we have termed calcium tsunamis. To our knowledge, this overwhelming calcium mishandling event has not been documented previously in human cardiomyocytes, likely because they only occur in large syncytia, such as those observed in this study, and not in single cells. The mechanism behind these large calcium release events requires further study. However, it is possible that calcium tsunamis are simply an extension of calcium waves which initiated by calcium sparks and propagated throughout the cell [32, 33]. We speculate that calcium handling abnormalities including calcium sparks, large “tsunami”-like waves, and increased calcium transient amplitude might directly alter L-type calcium channel function in the phase 2 and indirectly modify IKr/IKs in the phase 3 of action potential resulting in delayed repolarization.

We also found that CoV-2 S-mEm (spike protein)-induced hiPSC-CM syncytia display a marked reduction in repolarization reserve as evidenced by APD prolongation. Similarly, Marchiano et al. found that SARS-CoV-2 infection of hiPSC-CMs causes field potential duration (FPD) prolongation as measured by microelectrode array [22]. Prolongation of the APD, which is a cellular surrogate for QT prolongation in patients, is well known to cause cellular arrhythmias such as early afterdepolarizations in hiPSC-CMs and can cause the potentially fatal arrhythmia torsades de pointes in humans [29, 34]. Interestingly, SARS-CoV-2 infection causes an increase in baseline QTc in patients with COVID-19 [35]. Multiple potential explanations exist for this change including the presence of pro-inflammatory molecules such as IL-6 which causes a down-regulation of *KCNH2* expression leading to APD prolongation [36]. However, our data suggests that the presence of SARS-CoV-2 spike protein following infection of the heart could contribute directly.

Additionally, it has also been well documented that treatment with hydroxychloroquine and azithromycin, both of which are QT prolonging drugs, during the early pandemic caused cardiac complications and potentially fatal arrhythmias in numerous patients with COVID-19 [37, 38]. These drugs are historically very safe when used to treat patients with lupus or rheumatoid arthritis, but were eventually found to be not only ineffective, but also potentially dangerous, in the context of severe COVID-19 [37–41]. Further study is required to determine why this is, but our data points to the possibility that direct infection of the heart, along with the presence of pro-inflammatory molecules, may play a role in causing baseline QT prolongation thereby priming the heart for further drug induced QT prolongation and torsades de pointes following treatment with hydroxychloroquine and azithromycin.

Finally, Navaratnarajah et al. showed that treatment with a furin protease inhibitor and mutation of the furin cleavage site of CoV-2 S both prevented spike protein cleavage and prevented cell-cell fusion [23]. We were able to confirm these findings in our study and also showed that both methods restored calcium handling. Therefore, the combination of these studies may serve as the basis for the development of potentially novel cardioprotective agents which could be used to treat patients with COVID-19.

## Conclusions

Here, we found that the SARS-CoV-2 spike protein can directly produce cellular damage and electrophysiological dysfunction in hiPSC-CMs, and this may confer the intrinsic, mechanistic susceptibility for increased risk of SCD observed in patients with COVID-19. Additionally, our discovery that a furin inhibitor eliminates aberrant electrophysiology and hiPSC-CM dysfunction opens a new avenue in the development of cardioprotective agents.

## Supporting information

S1 Video Untransfected hiPSC-CMs display consistent rhythmic calcium transients. Video microscopy of Fluo-4 calcium imaging in untransfected hiPSC-CMs electrically paced at 1 Hz.
(MP4)

S2 Video Mock transfected hiPSC-CMs display consistent rhythmic calcium transients. Video microscopy of Fluo-4 calcium imaging in mock transfected hiPSC-CMs electrically paced at 1 Hz.
(MP4)

S3 Video SARS-CoV-2 spike protein-induced hiPSC-CM syncytia display calcium sparks. Video microscopy of Fluo-4 calcium imaging in CoV-2 S-transfected hiPSC-CMs electrically paced at 1 Hz. In this video, hiPSC-CMs are paced but display high levels of calcium sparks.
(MP4)

S4 Video SARS-CoV-2 spike protein-induced hiPSC-CM syncytia display calcium sparks and calcium tsunamis. Video microscopy of Fluo-4 calcium imaging in CoV-2 S-transfected hiPSC-CMs electrically paced at 1 Hz. In this video, hiPSC-CMs are paced but display high levels of calcium sparks and a calcium tsunami.
(MP4)

S5 Video SARS-CoV-2 spike protein-induced hiPSC-CM syncytia display loss of pacing, calcium sparks, and calcium tsunamis. Video microscopy of Fluo-4 calcium imaging in CoV-2 S-transfected hiPSC-CMs electrically paced at 1 Hz. In this video, hiPSC-CMs can no longer be paced and display high levels of calcium sparks and a calcium tsunami.
(MP4)

S1 Table Action potential parameters from untransfected and SARS-CoV-2S-mEmerald transfected hiPSC-CMs. (DOCX)

S1 Fig CoV-2 S resulted in significant syncytia formation compared with untransfected and Mock-transfection. Bar graph summary of the proportion of fused cardiomyocytes in untransfected, Mock-transfection, or CoV-2 S transfected cardiomyocytes.
(DOCX)
