# Supplementary material for: SARS-CoV-2 spike protein-mediated cardiomyocyte fusion may contribute to increased arrhythmic risk in COVID-19
Source: PLoS One. 2023 Mar 8;18(3):e0282151. doi: 10.1371/journal.pone.0282151 (PMC9994677; doi:10.1371/journal.pone.0282151)
Supplement: S1 Table — (DOCX) [file pone.0282151.s006.docx]

**Supplemental Table 1. Action potential parameters from** **untransfected and SARS-CoV-2S-mEmerald transfected hiPSC-CMs**

|  | Amp  (mV) | RMP  (mV) | MDP  (mV) | dV/dt_max_  (mV/ms) | APD20  (ms) | APD50  (ms) | APD90  (ms) |
| --- | --- | --- | --- | --- | --- | --- | --- |
| untransfected hiPSC-CMs  (n=10) | 83.1±2.3 | -49.0±3.2 | -60.4±1.8 | 16.5±2.7 | 241.2±21.0 | 352.1±24.9 | 419.3±25.7 |
| transfected hiPSC-CMs (n=10) | 94.8±3.9* | -51.4±2.8 | -63.7±2.4 | 12.5±2.9 | 443.5±77.3* | 536.4±68.6* | 589.8±66.5* |

*p<0.05 vs. untransfected hiPSC-CMs. Amp represents action potential amplitude, RMP represents resting membrane potential, MDP represents maximal diastolic potential and (dV/dtmax) represents maximal upstroke velocity. APD20, APD50 and APD90 represent the action potential duration at 20%, 50% and 90% repolarization.
