# Supplementary material for: SARS-CoV-2 spike protein-mediated cardiomyocyte fusion may contribute to increased arrhythmic risk in COVID-19
Source: PLoS One. 2023 Mar 8;18(3):e0282151. doi: 10.1371/journal.pone.0282151 (PMC9994677; doi:10.1371/journal.pone.0282151)
Supplement: S1 Fig — Bar graph summary of the proportion of fused cardiomyocytes in untransfected, Mock-transfection, or CoV-2 S transfected cardiomyocytes. (DOCX) [file pone.0282151.s007.docx]

**Supplemental Figure 1. CoV-2 S resulted in significant syncytia formation compared with untransfected and Mock-transfection.** Bar graph summary of the proportion of fused cardiomyocytes in untransfected, Mock-transfection, or CoV-2 S transfected cardiomyocytes.
